# Curettage of the Male Bladder for Chronic Cystitis1Read before the Surgical Section of the Rochester Academy of Medicine, April 11, 1900.

**Published:** 1900-06

**Authors:** Nathan W. Soble

**Affiliations:** Rochester, N. Y.


### CURETTAGE OF THE MALE BLADDER FOR CHRONIC CYSTITIS.[1]

REPORT OF A CASE.

By NATHAN W. SOBLE, M. D., Rochester, N. Y.

THE object of this short paper is to invite attention to the operation of curettage of the male bladder for chronic cystitis, and to report a case treated in that manner with favorable results. There are cases of chronic cystitis, nontubercular, that will not yield to any ordinary treatment. There are other cases that have gone so long untreated, or have been improperly treated, in which the ordinary methods in vogue will do no good.

1. Read before the Surgical Section of the Rochester Academy of Medicine, April 11, 1900.

It is not the reader's purpose to touch, at all, the usual modes of dealing with cystitis, except to say, that in those severe, persistent, seemingly incurable, pathological changes in the mucous membrane of the bladder, when medical treatment has failed, resort should be had to more radical surgical means. Drainage of the bladder, either by perineal or suprapubic routes, will cure some of these cases, but not all; it is in these cases that curettage should be done. Drainage, curettage and irrigation are advocated by many authorities, especially in France, where Guyon may be mentioned first.

Legnum, in the *Annual* for the genito-urinary diseases, advises these methods of treatment. In the same journal, June, 1897, Banzet says, "In chronic, persistent cystitis, in the male or female, curetting the bladder, followed by thorough irrigation and drainage, has yielded the best results."

Bardenheüser, in the *Centralblatt für Gynekologie*, No. 14, 1894, is even more radical, reports a case where he dissected away the entire mucous membrane of the bladder and claims that the vesicle mucosa is renewed as is the endometrium after curettement.

Cathcart, *British Med. Jour.*, October 5, 1895, claims to have originated the plan of drainage of the bladder by the exhaust pump method after suprapubic cystotomy.

Dawbarn, New York *Med. Jour.*, November 19, 1895, denies this and says that Dr. Swan, a dentist of Rochester, N.Y., originated this method and used it long before Cathcart.

It is found on searching the recent medical literature on this subject, that many authorities mention the operation of curettage of the bladder, especially in tubercular cystitis.

Case I.—D. D., German, aged 36, married eight years; one child; lithographer by occupation. First consulted the reader January 6, 1900. Present weight, 120 pounds. Usual weight, 170 pounds. No venereal disease; past health good. When twenty-three years old, had a congenital growth removed from the head of the penis in a hospital of Heidelberg. This operation resulted in a cicatricial contraction of the meatus urinarius. He was perfectly well up to July, 1895, when, after a picnic and having partaken of the usual refreshments—beer—he was attacked, for the first time, with sudden retention and pain in the region of the genitals. After suffering for some time, he was catheterised by a physician who evacuated three quarts of urine; this relieved him for three days, during which time he was able to pass urine, but with some difficulty. Then he gradually grew worse, when another physician did a meatotomy, after which he bled very profusely: when the wound healed he was much better and remained so for four months. He then became worse again, had frequent and painful urination, and passed blood often; he could not hold his urine but a short time and was unable to work. Then the meatus was again cut, and he was again relieved for a time, so that he could hold urine for three or four hours. Sounds were now passed for a short distance into the urethra, every third day, for the purpose of maintaining the caliber of the meatus.

While this treatment continued he was fairly comfortable. Then after a certain sounding, something seemed to "give way;" there was a sudden hemorrhage, after which the pain and trouble with urination all returned, and have remained constant ever since, for a period of two and one-half years. All treatment has been of no avail. At about this time a small gravel was passed. The history of this man's sufferings during the past two and one-half years is pitiable in the extreme.

*Status præsens.*—The patient is thin, pale and looks much worn from long suffering. Penis shows some hypospadias; meatus much contracted; has to urinate every twenty minutes day and night, and each attempt is attended with pain and stranguary; carries a catheter which he introduces and wears in order to get a little relief—without it he is in constant pain.

*Urinary Analysis.*—Alkaline, ammoniacal, thick with ropy mucus. Specific gravity, 1036. Albumin. No sugar. No casts. Blood, mucus and pus in abundance. Over 50 per cent. of the urine, by volume, is sediment and pus.

Only two ounces of water could be injected into the bladder without causing extreme pain. No prostatic enlargement. Repeated microscopical examinations made by Drs. Koch, Ruggles and the reader did not demonstrate any specific germs.

January 10, 1900.—Under ether the meatus easily dilated and a full-sized sound was passed into the bladder. No strictures below the meatus. Thompson's searcher passed easily and failed to find any stone, but the beak of the instrument seemed to rub against something soft. Capacity of bladder under ether, four ounces.

July 13, 1900, at St. Mary's Hospital, in the presence of Drs. Koch and Ruggles, suprapubic cystotomy was done and the bladder was stitched to the parietal peritoneum. Then the bladder was opened and inspected through the wound. The entire cavity of the bladder could be easily seen; there was no stone, and no tumor; the cavity was small; walls thickened and the mucous membrane apparently very red and shining, smooth in the main, though some rugosities could be felt with the fingers in the region of the trigone and internal opening of the urethra. The most striking feature was the extreme redness and inflamed appearance of the mucous membrane of the bladder.

For the next three days no urine was allowed to pass through the suprapubic incision; a catheter was fastened to the urethra. After that time the bladder was kept drained by syphonage and irrigated twice daily. It seemed remarkable that although nitrate of silver was injected into the bladder in fairly strong solution, up to 10 per cent., no pain was caused. Although the patient was rendered comfortable by the operation, obtaining rest and sleep with a consequent improvement in his general condition, there was not the slightest improvement in the condition of the bladder, which was examined frequently by the aid of a light, neither was there any change in the character of the urine—this remained as vile as before.

In spite of thorough drainage and frequent irrigation, up to February 9th there was no improvement in the condition of the bladder. On this date, the notes of the case state: no improvement in condition of urine; general condition of patient better; comfortable; temperature normal; external wound smaller; bladder irrigated by permanganate of potash, 1-2000, followed by sterile water twice daily. At this time it was decided to curette the mucous membrane of the bladder, through the suprapubic incision, for the purpose of removing what seemed to be a thickened, reddened and diseased epithelium, with the hope that a new, healthy epithelial layer would be formed.

The patient was anesthetised, the suprapubic opening dilated, and a small sharp uterine curette introduced. Extreme caution was exercised in the use of this instrument, very gentle force being used, and yet the curette penetrated into the wall of the bladder for a half inch or more; this could be done in any part of the bladder, the depth varying from a half inch to one and one-fourth inches in different portions of the bladder; the deepest places were in the most dependent portions of the baldder.

What at first seemed to be a highly inflamed epithelial layer, was in reality a plastic deposit, an exudate covering the entire mucous membrane of the bladder, rendered smooth and glistening by hydrostatic pressure. The exudate was thoroughly curetted and at the first sitting it could not be all cleaned out; the quantity seemed unlimited, and although the patient was kept under ether for nearly two hours, healthy bladder was reached only in portions. The bladder was then thoroughly irrigated. Hemorrhage was not particularly marked.

A second curettement was done ten days after the first. At this time healthy bladder wall was soon recognised by the sound of the curette scraping on healthy tissue. After the operation the cavity of the bladder was considerably increased and the patient began to improve at once. The urine became clear almost immediately and in three weeks' time was nearly normal. At the expiration of six weeks the external wound was healed, the patient was walking about, urinating every two hours during the day, and less often at night. He has gained thirty pounds in weight.

267 ANDREWS STREET.

